# Effect of treatment center volume on outcomes in gastroenteropancreatic neuroendocrine tumor patients

**DOI:** 10.1186/s12885-021-07868-8

**Published:** 2021-02-09

**Authors:** Kiwoon Baeg, Cynthia Harris, Monica S. Naparst, Eugene Ahn, Sahityasri Thapi, Jacob Martin, Sheila Rustgi, Grace Mhango, Juan Wisnivesky, Michelle Kang Kim

**Affiliations:** 1 Department of Medicine, Division of Gastroenterology, Icahn School of Medicine at Mount Sinai, 1 Gustave L Levy Place, New York, NY 10029 USA; 2 Department of Pathology, Massachusetts General Hospital, Boston, MA USA; 3 Department of Medicine, University of Pennsylvania, Philadelphia, PA USA; 4 Department of Medicine, Division of Internal Medicine, Icahn School of Medicine at Mount Sinai, New York, NY USA

**Keywords:** Gastroenteropancreatic neuroendocrine tumors, Carcinoid tumors, Center volume, Volume study, SEER Medicare

## Abstract

**Background:**

Medical centers with varying levels of expertise treat gastroenteropancreatic neuroendocrine tumors (GEP-NETs), which are relatively rare tumors. This study assesses the impact of center volume on GEP-NET treatment outcomes.

**Methods:**

We used the Surveillance, Epidemiology, and End Results (SEER) registry linked to Medicare claims data. The data includes patients diagnosed between 1995 and 2010 who had no health maintenance organization (HMO) coverage, participated in Medicare parts A and B, were older than 65 at diagnosis, had tumor differentiation information, and had no secondary cancer. We identified medical centers at which patients received GEP-NET treatment (surgery, chemotherapy, somatostatin analogues, or radiation therapy) using Medicare claims data. Center volume was divided into 3 tiers – low, medium, and high – based on the number of unique GEP-NET patients treated by a medical center over 2 years. We used Kaplan-Meier curves and Cox regression to assess the association between volume and disease-specific survival.

**Results:**

We identified 899 GEP-NET patients, of whom 37, 45, and 18% received treatment at low, medium volume, and high-volume centers, respectively. Median disease-specific survival for patients at low and medium tiers were 1.4 years and 5.3 years, respectively, but was not reached for patients at high volume centers. Results showed that patients treated at high volume centers had better survival than those treated in low volume centers (HR: 0.63, 95% CI: 0.4–0.9), but showed no difference in outcomes between medium and high-volume centers.

**Conclusions:**

Our results suggest that for these increasingly common tumors, referral to a tertiary care center may be indicated. Physicians caring for GEP-NET patients should consider early referral to high volume centers.

## Background

Gastroenteropancreatic neuroendocrine tumors (GEP-NETs) are rare solid tumors that originate from the cells of the neuroendocrine cell system throughout the gastrointestinal tract. Many studies have indicated that the incidence and prevalence of GEP-NETs is increasing globally [1–3]. A recent population-based study demonstrated a 6.4-fold increase in the incidence of neuroendocrine tumors between 1973 and 2012 based on data from the Surveillance, Epidemiology, and End Result (SEER) database [2]. This increase in incidence may be attributed to many factors, including improved availability and sensitivity of diagnostic modalities such as computed tomography and endoscopy [2, 4, 5]. As GEP-NET incidence also increases with age, this is a particularly important issue in an aging US population [2].

GEP-NETs frequently present with advanced stage and have significant morbidity and mortality from their disease [1, 4]. Depending on the location of primary tumor, treatment options may include endoscopic or surgical resection, hepatic embolization, and medical treatment with somatostatin analogues, interferon, and chemotherapy [6–8]. Furthermore, consensus on patient management is far from standardized due to the limited evidence available in the literature [9]. Treatments may vary between institutions, and recommendations often depend on local expertise as well as provider preference and experience [5].

Previous studies have demonstrated that improved clinical outcomes may be associated with high volume centers. One retrospective population-based study demonstrated that procedure volumes predict outcomes in surgical treatment of colon cancer [10]. Another study indicated that care at high-volume centers and from specialty physicians may be associated with improved long-term outcomes in ovarian, testicular, and breast cancer [11]. Furthermore, Begg et al. demonstrated that higher volumes were correlated with lower mortality in surgical cancer procedures such as pancreatectomy, esophagectomy, liver resection, and pelvic exenteration [12]. However, it remains unknown how center volume may affect outcomes in GEP-NET patients. We hypothesized that increased GEP-NET volume would be associated with improved clinical outcomes. Using a population-based cancer registry linked to insurance claims, we assessed the association between hospital volume and disease-specific survival (DSS) in elderly patients with GEP-NETs.

## Methods

### Data source and study population

For this study, we used the Surveillance, Epidemiology, and End Results (SEER) registry linked to Medicare claims [13]. SEER is a population-based registry detailing information on treatments and outcomes in cancer patients, while Medicare includes patients over the age of 65. The SEER-Medicare database involves the linkage of these two registries, therefore including patients over the age of 65 with cancer. The Institutional Review Board of the Mount Sinai School of Medicine approved this study. Our study cohort included patients diagnosed with a GEP-NET between January 1, 1995 and December 31, 2010. GEP-NET patients were identified using ICD-0-3 topographic codes (stomach C160-C169, duodenum C170, jejunum and ileum C171-C172, small intestine not otherwise specified C173-C179, cecum C180, appendix C181, colon C182-C189, rectum C199, C209, pancreas C250-C259), coupled with ICD-0-3 histology codes (8013, 8150–8157, 8240–8246, 8249, 8574, 9091). To ensure complete claims data, we only included patients who had exclusive Medicare coverage and participation in Medicare parts A and B for at least one-year post- diagnosis or until death, were 65 or older at time of diagnosis, and were not diagnosed on death certificates or autopsy. Patients with multiple malignancies were excluded to avoid misattribution of cancer treatment. Patients without tumor differentiation were excluded. Patients for whom the center of initial treatment could not be identified were also excluded.

We obtained demographic, staging, and differentiation data from SEER. Tumor stages were assigned according to SEER historic staging categories: local, regional, and distant. Tumor differentiation was coded as well, moderately, or poorly differentiated. Comorbidities were calculated using Charlson comorbidity indices with the Deyo modification [14]. HCPCS and ICD-O-9 codes on Medicare claims were used to assign patient treatment into 5 categories: surgery, somatostatin analogue, endoscopy, chemotherapy, and radiation therapy (Table 1).

**Table 1:** Characteristics of patients with Gastroenteropancreatic Neuroendocrine Tumors and of their initial treatments, SEER-Medicare 1995–2010; Small Intestine Not Otherwise Specified: SI NOS; National Cancer Institute: NCI

Characteristic	low (*n* = 331)	medium (*n* = 408)	high (*n* = 160)	*p*-value
Mean age (SD)	75.1 (7.3)	73.8 (7.1)	73.0 (6.2)	0.023
Median time to treatment (SD)	22.0 (47.5)	24.0 (41.8)	26.0 (55.1)	0.148
Male, n (%)	150 (45.3)	185 (45.3)	78 (48.8)	0.734
Race, n (%)				0.799
white	280 (84.6)	344 (84.3)	137 (85.6)	
black	26 (7.9)	38 (9.3)	> 12 (> 7.5)	
other	25 (7.6)	26 (6.4)	< 11 (< 6.9)	
Primary site, n (%)				<.0001
stomach	37 (11.2)	37 (9.1)	< 11 (< 6.9)	
duodenum	11 (3.3)	> 11 (> 2.7)	15 (9.4)	
jejunum/ileum	38 (11.5)	77 (18.9)	38 (23.8)	
SI NOS*	23 (7.0)	40 (9.8)	13 (8.1)	
appendix	< 11 (< 3.3)	< 11(< 2.7)	< 11 (< 6.9)	
cecum	71 (21.5)	71 (17.4)	13 (8.1)	
colon	80 (24.2)	77 (18.9)	25 (15.6)	
rectum	52 (15.7)	42 (10.3)	< 11 (< 6.9)	
pancreas	< 11 (< 3.3)	40 (9.8)	26 (22.5)	
Tumor stage, n (%)				0.0009
local	58 (17.5)	73 (17.9)	51 (31.9)	
regional	135 (40.8)	186 (45.59)	56 (35.0)	
distant	138 (41.7)	149 (36.5)	53 (33.1)	
Tumor differentiation, n (%)				<.0001
well-differentiated	82 (24.8)	167 (40.9)	107 (66.9)	
moderately-differentiated	70 (21.2)	74 (18.1)	19 (11.9)	
poorly-differentiated	179 (54.1)	167 (40.9)	34 (21.3)	
Comorbidity, n (%)				0.815
0	138 (41.7)	166 (40.7)	63 (39.4)	
1–2	142 (42.9)	169 (41.4)	73 (45.6)	
3+	51 (15.41)	73 (17.9)	24 (15.0)	
Treatment type, n (%)				<.0001
surgery	223 (67.4)	331 (81.1)	145 (90.6)	
endoscopy	21 (6.3)	16 (3.9)	< 11 (< 6.9)	
chemotherapy	25 (7.6)	20 (4.9)	< 11 (< 6.9)	
radiation therapy	12 (3.6)	< 11 (< 2.7)	< 11 (< 6.9)	
somatostatin analogue	35 (10.6)	14 (3.4)	< 11 (< 6.9)	
surgery and additional treatment(s)	15 (4.5)	> 16 (> 3.9)	< 11 (< 6.9)	
NCI designated cancer center*, n (%)				
yes	< 11 (< 3.3)	< 11 (< 2.7)	16 (10.0)	
no	308 (93.1)	383 (93.9)	123 (76.9)	
unknown	> 12 (> 3.6)	> 14 (> 3.4)	21 (13.1)	
				<.0001
Academic center*, n (%)				
yes	143 (43.2)	252 (61.8)	131 (81.9)	
no	173 (52.3)	> 145 (> 35.5)	> 18 (> 11.3)	
unknown	15 (4.5)	< 11 (< 2.7)	< 11 (< 6.9)	
				<.0001

*Small Intestine Not Otherwise Specified: SI NOS; National Cancer Institute: NCI

### Determining initial treatment records and center volume

We used the first treatment record found after a patient’s date of diagnosis to assign a center to each GEP-NET patient. For patients whose first treatment consisted of an inpatient or outpatient claim, center information was directly available. However, for patients whose first treatment record was a carrier claim, center information was not directly available. In these cases, the carrier claim’s center was determined by identifying the center at which the listed operating or attending physician practiced during that month. Inpatient and outpatient records were used to match the physician to a center. Remaining patients’ carrier claims’ centers were determined by matching the carrier claim date to the date in an inpatient or outpatient record. An error margin of 1 week was applied for this matching. We adopted this strategy to minimize error while ensuring that all patients could be linked to a medical center. For example, for a patient with date of service of January 1, 2020, we assessed inpatient and outpatient records from December 28, 2019 to January 4, 2020 to confirm association with a center and physician. This strategy allowed us to accurately link patients to a medical center via their treating physician.

Center volume was calculated as the number of unique single malignancy GEP-NET patients treated by a center 2 years prior to the treatment date of a cohort subject. The resulting center volumes were divided into three tiers: low (0–4 patients over 2 years), medium (5–14/2 years), and high volume (15+/2 years). The divisions were chosen in the way that produced the most even distribution between tiers. To assess for bias in the established volume tier divisions, we conducted sensitivity analysis by shifting boundaries between tiers by up to three and by implementing binary and quaternary tier divisions.

### Statistical analysis

Baseline demographic data were compared using χ^2^ and ANOVA analysis. Kaplan-Meier curves were constructed to evaluate differences in DSS among low, medium and high-volume centers. Log-rank tests were used to evaluate differences between tiers. We performed Cox proportional hazards analysis to compare DSS of patients treated in low, medium, and high-volume centers while adjusting for potential confounders. We adjusted for sex, primary site, treatment, comorbidity, differentiation, tumor stage, academic status, and National Cancer Institute (NCI) status. All analysis was done on SAS Studio (SAS Institute, Cary NC).

## Results

A total of 6124 GEP-NET patients participating in Parts A and B of Medicare within a year after diagnosis was identified in the SEER-Medicare database. We excluded 214 non-pathologically diagnosed, 2578 patients who were unable to be assigned to a center, 154 patients who were treated outside SEER areas, and 2279 patients without tumor differentiation. This resulted in a final cohort of 899 patients for this study.

The mean patient age was 75 years (standard deviation, 7). The population was 45% male and 85% white. Of the entire cohort, 20, 42, and 38% of the patients had local, regional, and metastatic disease stage, respectively. With respect to differentiation, 40, 18, and 42% had well-differentiated, moderately differentiated, and poorly/non-differentiated tumors. With respect to primary site, approximately 9% of NETs originated from the stomach, 30% from the small bowel 4% from the appendix, 49% from the colorectal region, and approximately 9% from the pancreas. The vast majority (77%) of these patients were surgically treated. The rest were treated with endoscopic excision, chemotherapy, somatostatin analogues, radiation therapy, or a combination of treatments.

Within our cohort, 37% were treated at low volume centers, 45% at medium, and 18% at high volume centers. Patients across the three tiers were comparable with respect to age, gender, race, and comorbidities (*p* > .05, Table 1). Patients treated at low volume centers more commonly had gastric (11% vs 9% medium, < 7% high) and colorectal NETs (61% vs 47% medium, 28% high), while patients treated at high volume centers more frequently had small bowel (41%, vs > 31% medium, 22% low) and pancreatic NETs (23% vs 10% medium, < 3.3% low; *p* < .0001). Patients treated at low volume centers more commonly had advanced stage disease (42%) and poorly differentiated tumors (54%) as compared to those treated at medium (37, 41%) and high-volume centers (33, 21%, *p* < .005). We found that 70% of patients with advanced stage disease and poorly differentiated tumors treated in low volume centers had colorectal NETs. Low volume center patients were least likely to undergo surgery as initial treatment (67%), as compared to those treated in medium and high-volume centers (81, 91%, *p* < .0001). In addition, low volume center patients received somatostatin analogue based treatments more often (10%) than those in medium or high-volume centers (3, < 7%, *p* < .0001).

**Fig. 1:**
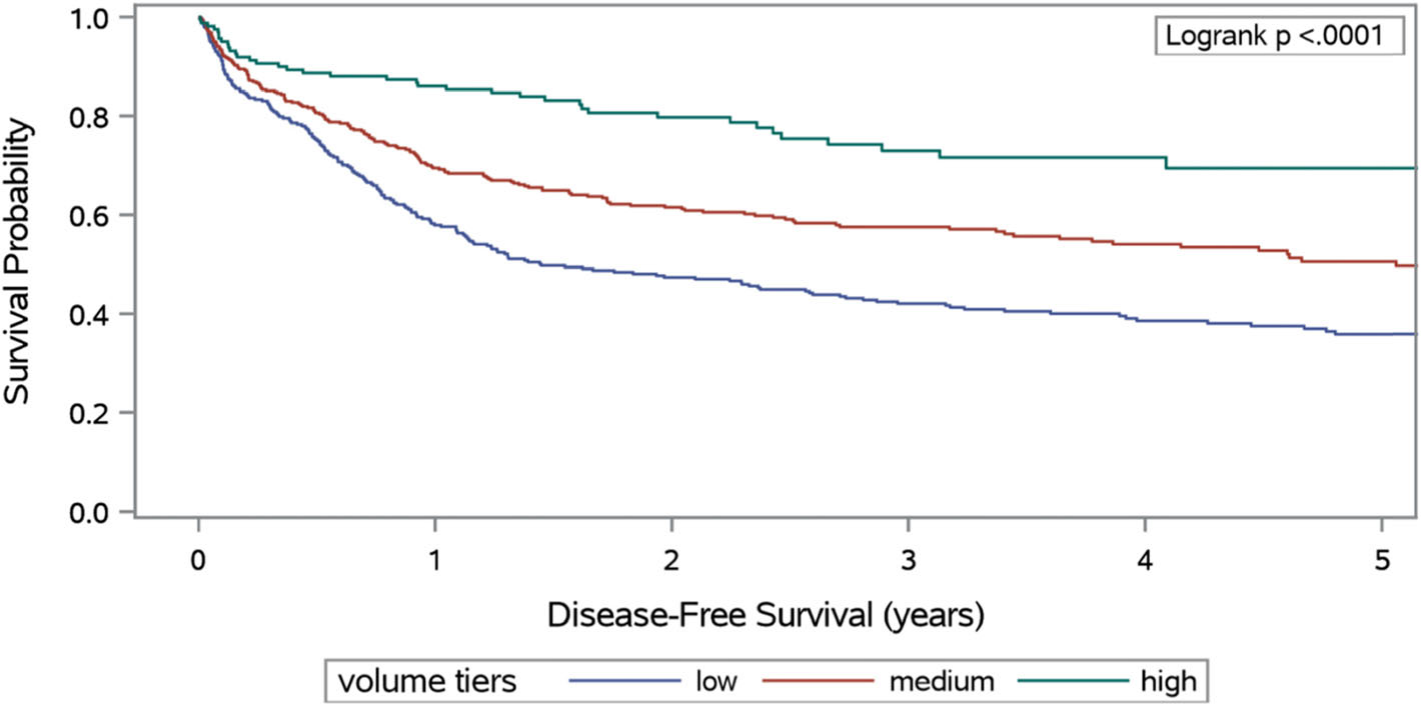
Disease-Specific Survival Following Date of GEP-NET Treatment, SEER-Medicare 1995–2010

Higher center volume was associated with improved DSS. Kaplan-Meier survival curves demonstrated median DSS for low and medium volume centers of 1.4 and 5.3 years, respectively, while median DSS for high volume centers was not reached (Fig. 1). DSS rates at 5 years were 36, 52 and 59% for low, medium, and high volumes. Log-rank tests indicated a statistically significant difference in DSS between the three tiers (*p* < .0001). After adjusting for gender, tumor related factors, and treatment, Cox proportional hazards analysis (excluding tier) confirmed the association between higher center volume and improved outcomes. Compared to low volume centers, medium and high-volume centers had 29% (HR: 0.69, 95%CI: 0.6–0.9) and 31% (HR: 0.63, 95%CI: 0.4–0.9) decreased hazards of death respectively (Table 2).

**Table 2:** Hazard Ratio (HR) and 95% Confidence Interval (CI) of Disease-Specific Survival Following Initial GEP-NET Treatment, SEER-Medicare 1995–2010; Small Intestine Not Otherwise Specified: SI NOS; National Cancer Institute: NCI

Variable	HR (95% CI)
Patient Volume	
low	Ref
medium	0.72 (0.57–0.91)
high	0.62 (0.42–0.94)
Sex	
female	Ref
male	1.33 (1.07–1.65)
Age	
65–69	Ref
70–74	1.29 (0.99–1.70)
75–79	1.23 (0.93–1.63)
80–84	1.24 (0.86–1.77)
85–89	2.03 (1.35–3.07)
90–94	2.10 (0.93–4.75)
Primary Site	
cecum	Ref
stomach	0.74 (0.49–1.11)
duodenum	1.10 (0.55–2.25)
jejunum/ileum	0.42 (0.27–0.65)
SI NOS*	0.54 (0.33–0.90)
appendix	0.73 (0.36–1.50)
colon	1.00 (0.73–1.35)
rectum	0.79 (0.56–1.12)
pancreas	0.88 (0.54–1.43)
Treatment	
surgery	Ref
endoscopy	0.87 (0.51–1.47)
chemotherapy	1.19 (0.81–1.75)
radiation therapy	2.72 (1.70–4.35)
somatostatin analogue	1.47 (1.04–2.10)
surgery and endoscopy	0.85 (0.46–1.57)
surgery and chemotherapy	0.91 (0.35–2.37)
surgery and radiation therapy	1.84 (1.13–2.98)
surgery, endoscopy, and chemotherapy	10.07 (6.91–14.674)
Comorbidity	
0	Ref
1–2	1.31 (1.05–1.64)
3+	1.70 (1.24–2.34)
Differentiation	
well-differentiated	Ref
moderately-differentiated	1.81 (1.28–2.58)
poorly-differentiated	3.82 (2.75–5.32)
Tumor Stage	
local	Ref
regional	1.76 (1.16–2.69)
distant	5.61 (3.72–8.46)
Academic status	
no	Ref
yes	1.20 (0.97–1.48)
NCI status	
no	Ref
yes	0.49 (0.18–1.30)

*Small Intestine Not Otherwise Specified: SI NOS; National Cancer Institute: NCI

Although there was a significant decrease (*p* < .05) in the DSS between medium and high-volume centers on Kaplan-Meier survival analysis, we no longer observed this difference after adjusting for multiple factors on multivariate Cox analysis. Instead, we noted an apparent two-tiered system where centers above a certain minimum volume appeared to be associated with improved outcomes. Sensitivity analysis conducted on tier boundaries revealed that the correlation between volume and DSS remained statistically significant as long as volume in low volume centers was less than four patients over 2 years. These results indicate that DSS was higher in centers that sustained a certain minimum volume of patients. However, our findings indicate that DSS did not necessarily improve past that point, as clinical outcomes seemed to plateau, even with higher center volumes.

## Discussion

Although GEP-NETs are an increasingly prevalent cancer, they are still a rare group of tumors with relatively low numbers of patients. Challenges persist in that there are few centers with specialists with strong expertise in this field. In this large population-based study, we found that medical center volume is associated with improved survival. This study is the first to look at the impact of medical center volume on patient outcomes. Our data suggests that regionalization of GEP-NET care could translate to improved outcomes and that a certain threshold of experience is needed to attain this benefit.

No previous studies have assessed the relationship between medical center volume and patient outcomes in NETs. Studies in more common cancers such as prostate and colorectal cancers have found that higher procedural volume is associated with improved survival [3, 10, 15]. Few studies, however, have focused on rarer forms of cancer (incidence rates < 6 in 100,000) [16]. Using the National Cancer Database, Chen et al. confirmed that laryngeal cancer patients experienced improved outcomes when administered surgical or radiation treatments at high volume centers [17]. In our study, we assessed the impact of volume on GEP-NETs, an equally rare cancer. In addition, our study looked not only at surgical/procedural volume, but more broadly at the entire clinical care of GEP-NET patients. This clinical care was often composed of multidisciplinary components including not only surgical treatments, but also radiation, chemotherapy, somatostatin analogues, and endoscopic procedures.

One interesting result was that high-volume centers were more likely to see patients with well-differentiated midgut and pancreatic NETs. Conversely, low volume centers were more likely to see poorly differentiated NETs, a large portion of which were of colorectal primary origin (72%). Although we might expect high numbers of patients with aggressive tumors to be seen in high-volume centers, one explanation is that perhaps the patients with well-differentiated and localized NETs are more likely to be recommended for surgical resection, and therefore more likely to be referred to a high-volume center.

Our study demonstrated that after adjustment for differences in tumor stage and grade, patients treated in higher volume centers had improved DSS over those treated in low volume centers. Sensitivity analysis confirmed the importance of center volume in influencing patient outcomes. We hypothesize that because GEP-NETs are relatively rare, have heterogeneous clinical courses, and diverse treat treatment options, GEP-NETs may require more expertise in choosing appropriate treatments. Therefore, an increased volume of patients may provide physicians with more experience, potentially leading to better clinical outcomes.

Our study had multiple strengths and limitations. This is the first study to assess NET patient outcomes with respect to medical center volume in an elderly population. Especially with an increasingly incident cancer in an aging U.S. population, these results provide evidence that NET patients will have improved outcomes in high volume centers. However, in order to have a cohort with complete insurance claims, a relatively large number of patients was excluded. Nevertheless, this study still reflects the experience of a large number of elderly GEP-NET patients. Another limitation is that patients were linked to a center based on where they were initially treated, and although most patients continued their care at the same center, some patients received subsequent care at a different medical center. This could have resulted in misattributing a patient’s outcome, whether favorable or poor, to the initial hospital. Another limitation is that we did not have information regarding tumor grade. In this study, however, we used tumor differentiation as a proxy for tumor grade.

To our knowledge, our study is the first to assess correlation of GEP-NET patient outcomes with hospital volume. The results of this study suggest that, regardless of primary tumor site, stage, and grade, centers with expertise in GEP-NET treatment demonstrate better patient outcomes. Interestingly, we also found that significantly more patients with higher grade tumors were treated at low volume centers, while patients with more common GEP-NETs (well differentiated, pancreas or midgut primaries, etc.) were more likely to be treated at a high-volume center.

In conclusion, patients treated at high volume centers experienced better survival than those in low volume centers. Our results suggest that centers with high volumes in these relatively rare tumors provide specialized experience that may impact care. Early referral to tertiary care centers should be considered by clinical care providers. Future studies should address the importance of hospital volume not only in initial treatment but also in overall treatment. Future studies should also assess how hospital volume impacts outcomes in younger patients with GEP-NETs.

## Data Availability

The data that support the findings of this study are available from the National Cancer Institute but restrictions apply to the availability of these data, which were used under license for the current study, and so are not publicly available. Data are however available from the authors upon reasonable request and with permission of the National Cancer Institute (https://seer.cancer.gov/).

## Abbreviations

DSS: Disease-specific survival
GEP-NET: Gastroenteropancreatic neuroendocrine tumor
HCPCS: Healthcare Common Procedure Coding System
HMO: Health maintenance organization
ICD-O-3: International Classification of Diseases for Oncology, third edition
SEER registry: Surveillance, Epidemiology, and End Results registry
